# Camalexin contributes to the partial resistance of *Arabidopsis thaliana* to the biotrophic soilborne protist *Plasmodiophora brassicae*

**DOI:** 10.3389/fpls.2015.00539

**Published:** 2015-07-21

**Authors:** Séverine Lemarié, Alexandre Robert-Seilaniantz, Christine Lariagon, Jocelyne Lemoine, Nathalie Marnet, Anne Levrel, Mélanie Jubault, Maria J. Manzanares-Dauleux, Antoine Gravot

**Affiliations:** ^1^UMR1349 IGEPP, INRALe Rheu, France; ^2^Plateau de Profilage Métabolique et Métabolique (P2M2), Centre de Recherche Angers Nantes BIA, INRA de RennesLe Rheu, France; ^3^UMR1349 IGEPP, Agrocampus OuestRennes, France; ^4^UMR1349 IGEPP, Université de Rennes 1Rennes, France

**Keywords:** clubroot, partial resistance, phytoalexin, camalexin, *Arabidopsis thaliana*, *Plasmodiophora brassicae*, quantitative trait loci

## Abstract

Camalexin has been reported to play defensive functions against several pathogens in *Arabidopsis*. In this study, we investigated the possible role of camalexin accumulation in two *Arabidopsis* genotypes with different levels of basal resistance to the compatible eH strain of the clubroot agent *Plasmodiophora brassicae*. Camalexin biosynthesis was induced in infected roots of both Col-0 (susceptible) and Bur-0 (partially resistant) accessions during the secondary phase of infection. However, the level of accumulation was four-to-seven times higher in Bur-0 than Col-0. This was associated with the enhanced transcription of a set of camalexin biosynthetic P450 genes in Bur-0: *CYP71A13, CYP71A12*, and *CYP79B2*. This induction correlated with slower *P. brassicae* growth in Bur-0 compared to Col-0, thus suggesting a relationship between the levels of camalexin biosynthesis and the different levels of resistance. Clubroot-triggered biosynthesis of camalexin may also participate in basal defense in Col-0, as gall symptoms and pathogen development were enhanced in the *pad3* mutant (Col-0 genetic background), which is defective in camalexin biosynthesis. Clubroot and camalexin responses were then studied in Heterogeneous Inbred Families (HIF) lines derived from a cross between Bur-0 and Col-0. The Bur/Col allelic substitution in the region of the previously identified clubroot resistance QTL *PbAt5.2* (Chromosome 5) was associated with both the enhanced clubroot-triggered induction of camalexin biosynthesis and the reduced *P. brassicae* development. Altogether, our results suggest that high levels of clubroot-triggered camalexin biosynthesis play a role in the quantitative control of partial resistance of *Arabidopsis* to clubroot.

## Introduction

Clubroot is a disease that occurs worldwide in all *Brassicaceae* species, and causes important agronomic damage to *Brassica* crops, especially *B. napus, B. rapa*, and *B. oleracea* (Dixon, 2009). The infection is characterized by an asymptomatic primary phase, where germinated resting spores infect root hairs, followed by a secondary phase where plasmodia progressively develop inside the root cortex and stele cells. This secondary phase, which typically develops over 2–5 weeks in *A. thaliana*, is associated with hyperplasia and hypertrophy of plant host cells, resulting in the formation of root galls (Kageyama and Asano, 2009). In *Arabidopsis*, we previously reported that the Bur-0 accession harbors quantitative partial resistance against the telluric agent of clubroot, *Plasmodiophora brassicae* (Alix et al., 2007). Four additive QTLs (*PbAt1, PbAt4, PbAt5.1*, and *PbAt5.2*) were involved in the quantitative resistance of this accession (Jubault et al., 2008), and we previously demonstrated that the QTL *PbAt5.1* was associated with the ability to tolerate exogenous trehalose (Gravot et al., 2011). In a preliminary screen to identify defense response patterns triggered by clubroot infection, we also observed that one of the most prominent features of the Bur-0 response to clubroot is high-levels of camalexin (data not published).

**Figure 1:**
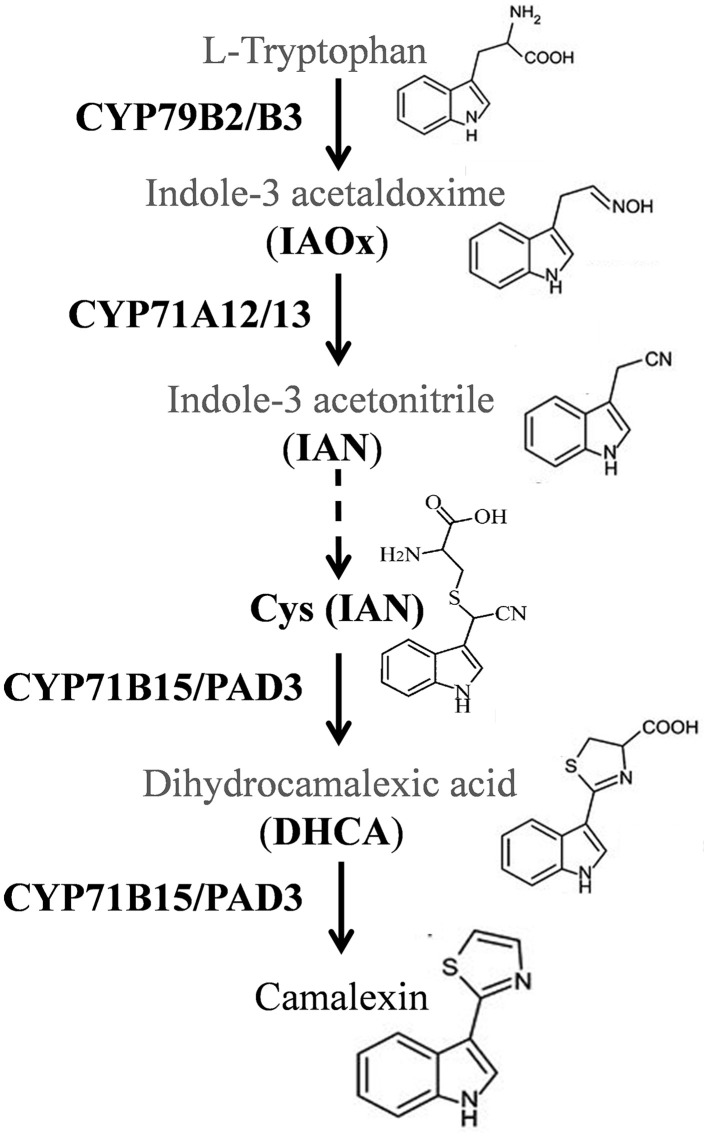
**Camalexin biosynthesis pathway according to Millet et al. (2010) and Geu-Flores et al. (2011)**. Camalexin is derived from tryptophan which is firstly converted to indole 3 acetaldoxime (IAOx) by the cytochrome P450 enzymes CYP79B2 and CYP79B3. IAOx is then converted to indole 3 acetonitrile (IAN) by the CYP71A12 and CYP71A13 enzymes. Subsequently, IAN is converted to the Cys(IAN) conjugate after intermediate steps (represented in dashed arrows). The final two steps in camalexin biosynthesis are catalyzed by the P450 enzyme CYP71B15/PAD3.

Camalexin is a sulfur-containing tryptophan-derived secondary metabolite, and is considered to be the major phytoalexin involved in biotic responses in *A. thaliana* (Ausubel et al., 1995; Glawischnig, 2007). The camalexin biosynthesis pathway (summarized in Figure 1) first involves the conversion of tryptophan to indole-3-acetaldoxime (IAOx), through the action of two functionally redundant cytochrome P450 enzymes, CYP79B2 and CYP79B3. This step is followed by the dehydration of IAOx to indole 3 acetonitrile (IAN), catalyzed by CYP71A13 (Nafisi et al., 2007) and CYP71A12 (Millet et al., 2010; Saga et al., 2012). IAN is then conjugated to glutathione by the glutathione-S-transferase GSTF6 to synthesize GSH(IAN) (Su et al., 2011) then metabolized to Cys(IAN) by γ-glutamyl peptidases GGP1 and GGP3 (Geu-Flores et al., 2011). Finally, the PAD3/CYP71B15 enzyme catalyzes the last two reactions of the biosynthesis pathway leading to camalexin (Zhou et al., 1999; Schuhegger et al., 2006; Böttcher et al., 2009). Many genetic approaches confirmed that camalexin plays a positive role in resistance. For instance, camalexin accumulation was correlated with resistance to necrotrophic fungi such as *Alternaria brassicicola* (Thomma et al., 1999; Nafisi et al., 2007), *Botrytis cinerea* (Ferrari et al., 2003, 2007; Kliebenstein et al., 2005; van Baarlen et al., 2007) and *Plectosphaerella cucumerina* (Staal et al., 2006; Sanchez-Vallet et al., 2010). Camalexin has also been reported to play a defensive role against the hemibiotrophic fungus *Leptosphaeria maculans* (Bohman et al., 2004; Staal et al., 2006) and the oomycete *Phytophthora brassicae* (Schlaeppi et al., 2010). However, Camalexin accumulation was not always correlated with pathogen resistance. For example, camalexin accumulated in response to various strains of *Pseudomonas syringae*, but the *pad3* mutant, in which the last two steps of camalexin biosynthesis are disrupted, did not show any difference in susceptibility to those strains (Glazebrook et al., 1997; Zhou et al., 1999).

Clubroot-induced camalexin accumulation was previously reported in Col-0 and several other *Arabidopsis* accessions (Siemens et al., 2008). The absence of enhanced clubroot susceptibility in the *pad3* mutant led the authors to conclude that camalexin was not likely to play a role in clubroot resistance. However, our preliminary assays indicated that camalexin levels accumulate at high levels in the partially resistant accession Bur-0 compared to the susceptible accession Col-0. Thus, we carried out an in depth investigation of the role of camalexin in the defense response toward *P. brassicae* in *A. thaliana* in those accessions. We first evaluated the time-course of camalexin accumulation and camalexin biosynthesis gene expression during clubroot infection in Bur-0 and Col-0. We also followed pathogen growth dynamics using a combination of histological and PCR-based pathogen quantification, over the same time-course. Thus, the time-course of camalexin accumulation and post-invasive partial resistance establishment could be compared. The role of clubroot-triggered camalexin biosynthesis in Col-0 was reassessed by phenotyping the *pad3* mutant challenged with the eH isolate. Finally, we tested possible genetic links between two major partial resistance QTL from Bur-0, *PbAt1*, and *PbAt5.2*, and the intensity of the clubroot-induced camalexin response. For this purpose, the time-course of pathogen development, camalexin accumulation and camalexin-biosynthesis gene transcription was evaluated in pairs of appropriate near isogenic Heterogeneous Inbred Lines (HIF) developed from the Bur-0 × Col-0 cross.

## Materials and methods

### Inoculum and plant material

The inoculum used in all the clubroot tests was the “selected” eH isolate (Fähling et al., 2003) which belongs to the pathotype P1 according to Somé et al. (1996). The host differential set described by Somé et al. (1996) was included in each test as a control.

The Versailles *A. thaliana* Resource Centre provided all the *Arabidopsis* seeds used in the study. The Bur-0 (172AV) and Col-0 (186 AV) accessions were described previously as partially resistant and susceptible to the eH isolate respectively by Alix et al. (2007). Genetic analysis of a Recombinant Inbreed Line (RIL) population generated from the Bur-0 × Col-0 cross led to the detection of four additive and two epistatic QTLs conferring partial resistance to clubroot in the Bur-0 accession (Jubault et al., 2008).

Heterogeneous Inbred Families (HIF) pairs were obtained from the Versailles *Arabidopsis* Stock Centre (publiclines.versailles.inra.fr). The HIFs were derived from RIL lines described in Simon et al. (2008). The HIF pair 499 shows polymorphism at the QTL *PbAt5.2*, harboring either the Col-0 susceptibility or the Bur-0 resistance allele. This HIF pair harbors the Bur-0 resistance allele at the QTL *PbAt1.* The HIF pair 508 shows polymorphism at the QTL *PbAt1*, harboring either the Col-0 susceptibility or the Bur-0 resistance allele. This HIF pair harbors the Bur-0 resistance allele at the QTL *PbAt5.2* (Supplementary Figures S2, S3).

Dr. Erich Glawischnig (Technische Universität, München) kindly provided the seeds of the camalexin deficient homozygous *phytoalexin deficient 3* (*pad3*) T-DNA mutant (SALK_026585).

### Clubroot tests and symptom quantification

Clubroot symptoms were quantified for four biological replicates, each containing 12–18 plants per genotype. Seeds of the susceptible Col-0, the partially resistant Bur-0 and the HIF pairs 499 and 508 were sown individually in “Mottefertiss” pots containing a mix of compost:vermiculite (2:1, v/v). Seedlings were grown in a growth chamber (16 h of light at 22°C at 200 μmol m^−2^ s^−1^ and 8 h of dark at 19°C) and were inoculated at the crown 10 days after germination with 1 ml of the eH spore suspension (10^7^ spores ml^−1^) (Manzanares-Dauleux et al., 2000) or distilled water for non-inoculated plants.

The susceptibility of plants to clubroot was evaluated at 17 and 21 days post-inoculation (dpi) by symptom quantification using image analysis. Inoculated plants were washed and photographed with a scale and symptoms were evaluated using the GA/LA pathological index. Briefly, this index was calculated from the ratio between the gall area (GA in cm^2^) and the square of the longest leaf length of the rosette (LA in cm^2^), determined by ImageJ software, which was then multiplied by 5000 (Gravot et al., 2011). After being photographed, 3 cm of roots was collected from all plants, pooled, frozen in liquid nitrogen and stored at −80°C for molecular and biochemical analysis.

### Pathogen DNA quantification by real-time PCR in infected roots

Total genomic DNA was extracted from 50 mg (12–54 freeze dried pooled plants depending on the sampling time) of infected roots (10, 14, and 17 dpi) using the “NucleoSpin Plant II” kit (Macherey-Nagel) following the manufacturer's instructions. The DNA quality was verified on agarose gel and the quantity was estimated with a ≪ Nanodrop 2000 ≫ (Thermoscientific). The final DNA concentration was adjusted to 10 ng/μL for each sample. Semi quantitative real-time PCR was performed in a Light Cycler 480 thermocycler (Roche) in a 12.5 μl volume with the following components: 2.5 ng of DNA, 6.25 μL of 2X Light Cycler 480 Syber Green I Master (Roche), 4 mM of forward and reverse primers and 1.25 μl of ultrapure water. The *Arabidopsis* F-box protein gene (At5g15710) previously described by Czechowski et al. (2005) was used to normalize the results and the *P. brassicae* target gene [part of the 18 s region (AF231027)] was previously described by Faggian et al. (1999). The primer sets used were as follows: Pb F, 5′-AAACAACGAGTC AGCTTGAATGC-3′; Pb R, 5′-AGGACTTGGCT GCGGATCAC-3′; *F-Box* F, 5′-TTTCGGCTGAGA GGTTCGAGT-3′; *F-Box* R, 5′-GATTCCAAGACGT AAAGCAGATCAA -3′. Quantitative PCR reactions were carried out with 50 cycles of denaturation at 95°C for 15 s and annealing/extension at 61°C for 30 s, followed by melt curve analysis. Amplification specificity was assessed by both melt curve analyses and agarose gel electrophoresis. Four biological replicates were analyzed for each time point. The results were expressed as the ratio between the DNA quantities of *P. brassicae* and the corresponding plant genotype DNA multiplied by 100.

### Histology

Infected and non-infected roots collected at 17 dpi were fixed in a glutaraldehyde (2%) and paraformaldehyde (1%) phosphate buffer (0.1 M pH 7.2), then washed with phosphate buffer (0.1 M pH 7.2) and distilled water, dehydrated in different ethanol:water solutions (10, 30, 50, 70, 90, and 100%) and finally embedded in resin with the Technovit 7100 kit (Heraeus Kulzer). 4 μm thick histological sections were cut with a microtome (Microm Microtech) and stained in cotton blue (1%) and safranin (1%) to differentiate the pathogen plasmodia and the root plant cells respectively during microscopic investigations. The impact of the infection on xylem vessel upkeep was visualized with an epifluorescence Nikon Eclipse E200 microscope (BP 365 nm, LP400 nm) after staining the sections with aniline blue (0.5%) dissolved in lactophenol.

### RNA extraction and real-time RT-PCR

Total RNA from 10, 14, and 17 dpi infected and non-infected roots was extracted using the ≪ SV Total RNA Isolation System ≫ kit (Promega) according to the manufacturer's instructions, with an additional DNAse step using the ≪ Ambion DNA-free ≫ kit (Ambion). First strand cDNA was synthesized in a 20 μl reaction mixture containing 1.6 μg of treated total RNA with the ≪ Superscript II Reverse Transcriptase ≫ kit (Invitrogen) with oligo-(dT)_15_ primers following the manufacturer's instructions. Semi-quantitative real-time PCR reactions were performed as follows in a 12.5 μl final volume: 4 μl of diluted cDNA, 6.25 μl of 2X Light Cycler 480 Syber Green I Master (Roche), 1.25 μl of nuclease free water and 4 mM of forward and reverse primers. The primer sets used to analyze the expression of the camalexin biosynthesis genes were as follows: *CYP79B2* (At4G39950) F, 5′-CCACTGCAACCGA AACATCG-3′; *CYP79B2* R, 5′-GGCTCTTTAGCATCGT CGGA-3′; *CYP79B3* (At2G22330) F, 5′-CTCTTCGGATCTC ACGACCA-3′; *CYP79B3* R, 5′-CATCAAGAAGCAAAGGGC CG-3′; *CYP71A12* (At2G30750) F, 5′-TCCCAAGCGATGTT ACGAGT-3′; *CYP71A12 R*, 5′-CTGTCTATCCATGCCAAAG CC-3′; *CYP71A13* (At2G30770) F, 5′-GCCCCGGGATAAATC TTGCT-3′; *CYP71A13* R, 5′-TGTTGCATAGCATAACAA GGTGA-3′; *PAD3/CYP71B15* (At3G26830) F, 5′-GGAGTC GCTGGCATAACACT-3′; *PAD3/CYP71B15* F, 5′-ATGT CTCCTTGACCACGAGC-3′ and the housekeeping gene *PP2A* (At1G13320) F, 5′-TAACGTGGCCA AAATGATGC-3′; 5′-GTTCTCCACAA CCGCTTGGT-3′ described by Czechowski et al. (2005). Amplification reactions were carried out with 50 cycles of denaturation at 95°C for 15 s, annealing/extension at 60°C for 30 and 72°C for 30 s, respectively, followed by melt curve analysis. CP values were obtained for each gene studied and converted to arbitrary units. The final results were expressed as the ratio between the gene of interest and the housekeeping gene in arbitrary units. Two technical and three biological replicates were analyzed.

### Camalexin quantification

The accumulation of camalexin in infected and non-infected roots of each genotype was determined at 10, 14, and 17 dpi. For each time point and genotype, camalexin was extracted from approximately 200 mg of freshly ground roots in 1.5 mL tubes. After addition of 1 mL of a methanol:water:formic acid (80:19:1) (v:v:v) mixture solvent, tubes were ultrasonicated and agitated at room temperature for 30 min. The tubes were then centrifuged at 1200 g for 10 min and the supernatants were removed into new 1.5 mL tubes. The pellets were re-extracted with 1 ml of the extraction solvent and the supernatants were pooled with those from the first extraction and dried in a speed vacuum centrifuge. Dried residues were then resuspended in 100 μl of acidified methanol and 5 μl were injected and analyzed on an Acquity UPLC system (Waters) coupled to a Quattro Premier XE equipped with an electrospray ionization (ESI) source. Chromatographic separation was performed on an Acquity HSS C18 T3 1.8 μm (2.1 × 150 mm) column using a gradient of two mobile phases corresponding to an A solution (0.1% of formic acid and water) and B solution (0.1% of formic acid and methanol). The elution gradient started with 99% of A and 1% of B, then 20 min later 100% of B and returned to the initial conditions 25 min after the start of the elution. This separation step was at 40°C with a flow rate of 0.35 ml min^−1^ and the retention time of the camalexin was determined at 12.77 min. The eluted camalexin was ionized in negative mode at the ESI source of the mass spectrometer and fragmented at 40 V. Data were acquired in Multi Reaction Monitoring (MRM) mode, using the transition 199 > 141, with Masslynx software and results were expressed by reporting MS peak areas of the corresponding camalexin concentration in ng mL^−1^ determined using a camalexin standard (kindly provided by Pr P. Simoneau, University of Angers).

### Statistical analysis

Statistical analyses were performed with R software by using Wald tests applied on Linear Mixed Models (function “lme,” package “nlme”). Each model took into account the genotype, the kinetic time point of sampling and the inoculation as fixed factors and biological replicates as random factors. When needed, pairwise comparisons of Least Squares Means were computed (function “lsmeans,” package “lsmeans”). The alpha level was set at a standard level of 5%.

## Results

### Time-course of camalexin accumulation in Col-0 and Bur-0 during clubroot infection

**Figure 2:**
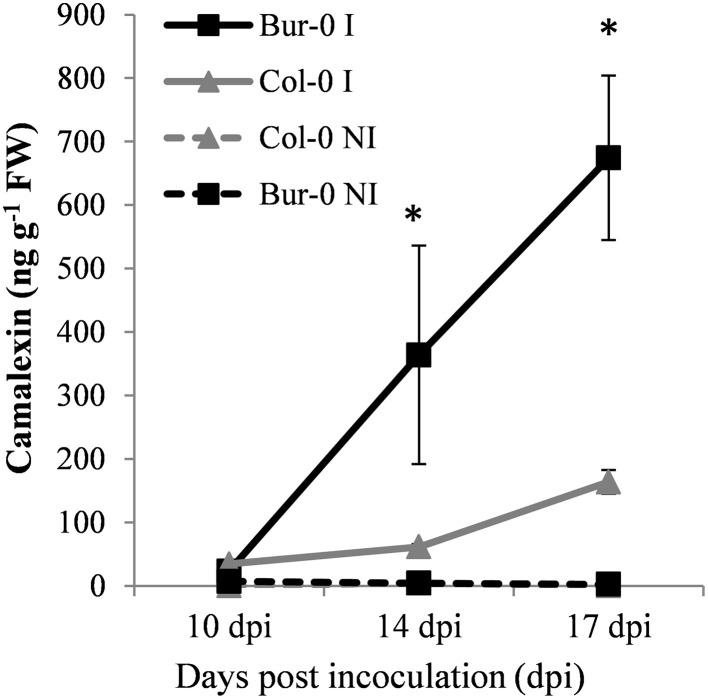
**Camalexin content in infected (continuous lines) and non-infected (dashed lines) roots of the partially resistant accession Bur-0 and the susceptible accession Col-0 at 10, 14, and 17 dpi**. Camalexin was quantified in root methanol extracts using UPLC-MS/MS, and is expressed as ng g^−1^ of fresh weight. Error bars represent standard error (three biological replicates, 12–54 plants analyzed per biological replicate). Asterisks indicate statistically significant differences according to the Wald tests applied on a linear mixed model (*P* < 0.05).

Camalexin was initially identified as a promising metabolic marker of clubroot resistance in a preliminary assay in which HPLC-MS profiles of defense compounds triggered by clubroot infection in Bur-0 and Col-0 were determined (data not shown). Consequently, camalexin levels were accurately quantified—using an UPLC-MS/MS method coupled with an authentic chromatographic chemical standard—in non-infected and infected roots of Col-0 and Bur-0 at different times during the secondary phase of infection. The results showed that the camalexin concentration was very low in non-infected roots (Figure 2). At 10 dpi, the camalexin content showed a weak increase in infected roots of both plant genotypes with no significant differences between Col-0 and Bur-0 at this time point (*P* = 0.060). At 14 dpi, the camalexin content in infected roots increased in both genotypes and was seven times higher in the partial resistant Bur-0 genotype than in the susceptible Col-0. At 17 dpi, the camalexin content was again enhanced in the infected roots of both genotypes, and reached more than four times higher levels in Bur-0 than in Col-0.

### Transcriptional regulation of the camalexin biosynthetic pathway in Bur-0 and Col-0 during clubroot infection

**Figure 3:**
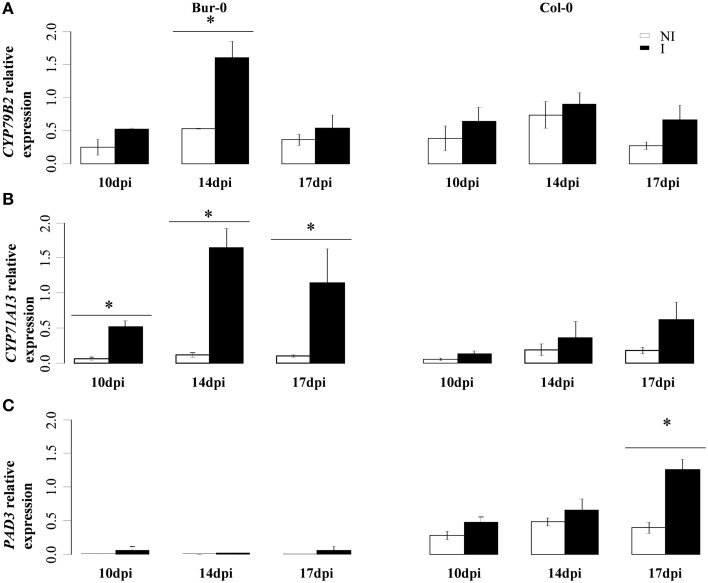
**(A)** Transcript levels of *CYP79B2*, **(B)** *CYP71A13*, and **(C)** *PAD3* in infected (black bars) and non-infected roots (white bars) of the partially resistant accession Bur-0 and the susceptible accession Col-0 at 10, 14, and 17 dpi. **(A–C)**, Expression levels were normalized using the reference gene *PP2A*. Error bars represent standard error (four biological replicates, 12–54 plants analyzed per biological replicate). Asterisks indicate statistically significant differences according to the Wald tests applied on a linear mixed model (*P* < 0.05).

Quantitative RT-qPCR analyses were performed to evaluate the transcriptional regulation of four camalexin biosynthesis genes in both Col-0 and Bur-0 accessions: *CYP79B2, CYP71A13, CYP71A12*, and *CYP71B15/PAD3*, over the infection time-course (Figure 3). *CYP79B2* encodes a P450 involved in the first biosynthetic step (tryptophan to indole-3-acetaldoxime conversion). Clubroot infection induced its expression in Bur-0 at 14 dpi (Figure 3A). *CYP71A13* encodes a P450 involved in Indole-3-acetaldoxime to Indole-3-acetonitrile dehydration. It showed stable expression in non-inoculated roots but was significantly upregulated in clubroot infected Bur-0 at all the time points studied (10, 14, and 17 dpi) (Figure 3B). In infected Col-0, *CYP71A13* induction was not statistically significant in our experimental conditions despite an apparent upward trend at 17 dpi (Figure 3B). Clubroot infection also induced the closely related P450 *CYP71A12*, involved in this same biochemical step, at both 14 and 17 dpi, with a higher level of induction in Bur-0 (Supplementary Figure S1). The basal expression levels of *CYP71B15/PAD3* (encoding the single P450 enzyme involved in the two last steps of camalexin biosynthesis) were lower in Bur-0 than in Col-0 in non-inoculated roots. This gene was not significantly induced by clubroot infection in Bur-0 and was induced at 17 dpi in infected Col-0 roots (Figure 3C).

### The camalexin-deficient mutant *PAD3* more susceptible to *P. brassicae* than Col-0

**Figure 4:**
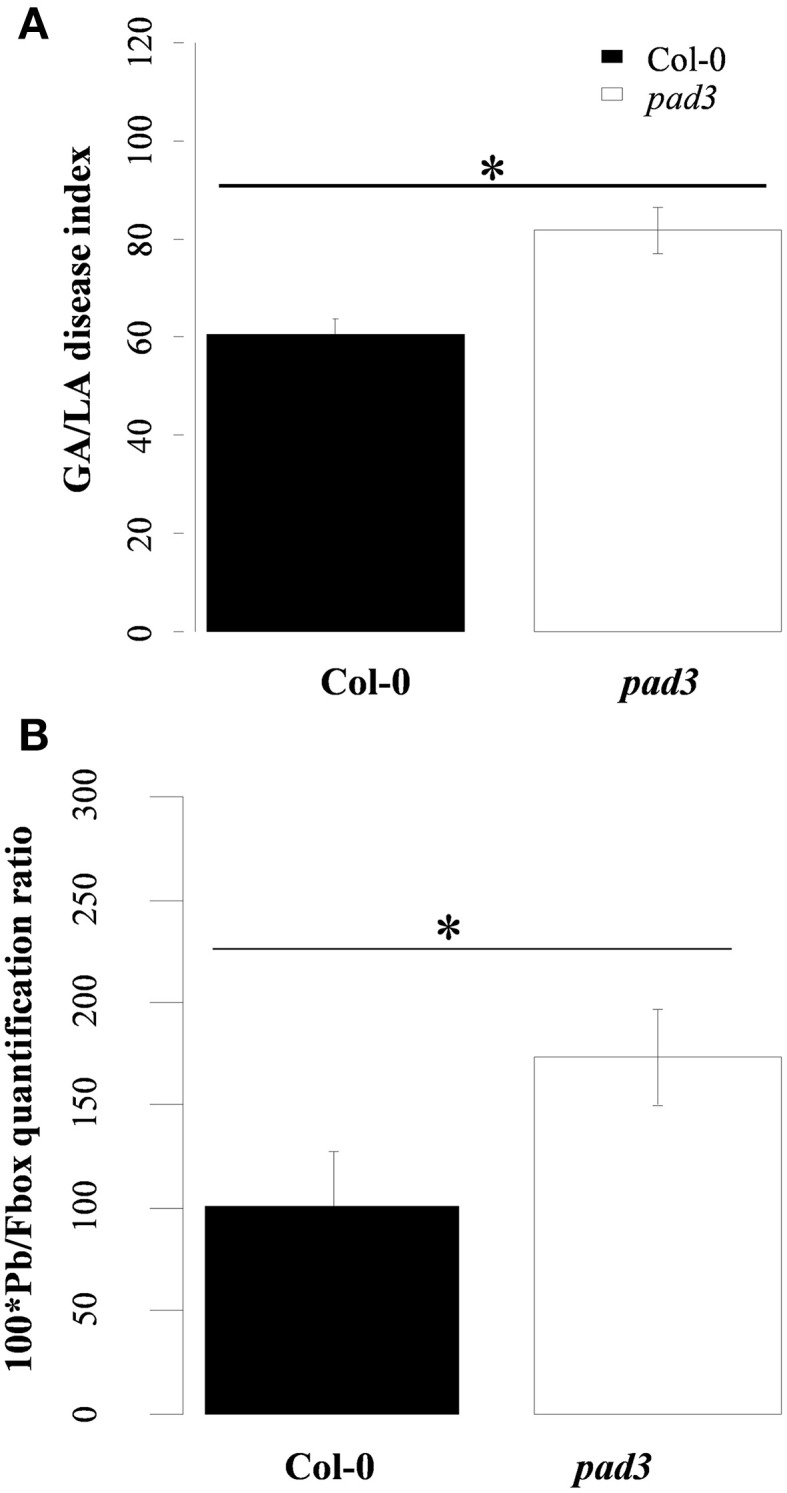
**(A)** Clubroot symptoms and **(B)** quantification of *Plasmodiophora brassicae* DNA in infected roots of the clubroot susceptible WT Col-0 and *pad3*. **(A)** Clubroot symptoms were evaluated using the GA/LA disease index calculated by image analysis at 21 dpi. GA/LA is the ratio between gall area (GA in cm^2^) and an estimation of the rosette extent (LA in cm^2^). Error bars represent standard error (Four biological replicates, six plants per biological replicate). **(B)** Pathogen DNA quantification (Pb) by qPCR, expressed as a ratio relative to the expression level of the plant *Fbox* gene, at 21 dpi (Four biological replicates, six plants per biological replicate). Asterisks indicate statistically significant differences according to the Wald tests applied on a linear mixed model (*P* < 0.05).

Although it accumulated at lower levels than in Bur-0, as described above, there was significant clubroot-triggered biosynthesis of camalexin in Col-0 at 17 dpi. Thus, we evaluated whether, under our experimental conditions, this camalexin accumulation is involved in the control of post-invasive basal resistance to the eH isolate. To test this hypothesis, clubroot symptoms and root pathogen content were evaluated in the *pad3* mutant (Col-0 background). The results are shown in Figure 4 and clearly indicated that, at 21 dpi, both symptom severity and pathogen content in infected roots were enhanced in *pad3* compared to the wild type Col-0. This suggests that the camalexin response does contribute to a late and weak basal control of clubroot symptoms and pathogen development in Col-0.

### *P. brassicae* growth was slower in Bur-0 than in Col-0 during the secondary phase of infection

**Figure 5:**
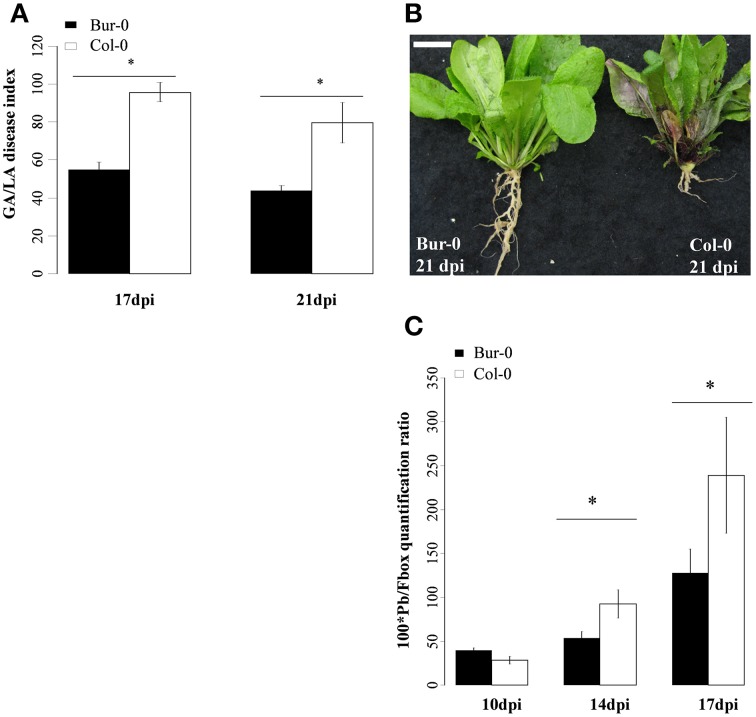
**(A,B)** Clubroot symptoms and **(C)**, quantification of *Plasmodiophora brassicae* DNA in infected roots of the partially resistant accession Bur-0 and the susceptible accession Col-0. **(A)** Clubroot symptoms were evaluated using the GA/LA disease index calculated by image analysis at 17 and 21 dpi. GA/LA was calculated from gall area (GA in cm^2^) divided by an estimation of the rosette extent (LA in cm^2^). Error bars represent standard error (Four biological replicate, 18 plants analyzed per biological replicate). Asterisks indicate statistically significant differences according to the (*P* < 0.05) **(B)** Illustration of clubroot symptoms. The scale bar indicates 1 cm. **(C)** Pathogen DNA quantification (Pb) by qPCR, expressed as a ratio relative to the expression level of the plant *Fbox* gene at 10, 14, and 17 dpi (Three biological replicates, 12–54 plants per biological replicate). Asterisks indicate statistically significant differences according to the Wald tests applied on a linear mixed model (*P* < 0.05).

We then compared symptom development and pathogen growth with the time-course of camalexin accumulation in both genotypes. Disease symptoms were quantified at 17 and 21 dpi and showed a two-fold increase in the severity of clubroot symptoms in Col-0 compared to Bur-0 at both time points (Figures 5A,B). The ratio between pathogen and plant DNA content was determined in infected Col-0 and Bur-0 roots. At 10 dpi, no significant difference in relative pathogen DNA content between the two genotypes was observed (*P* = 0.134). At 14 and 17 dpi, the relative pathogen DNA content increased in both genotypes, but to a higher degree in Col-0 than in Bur-0. Thus, pathogen DNA content was two-times higher in Col-0 than in Bur-0 infected roots at 14 and 17 dpi (Figure 5C).

**Figure 6:**
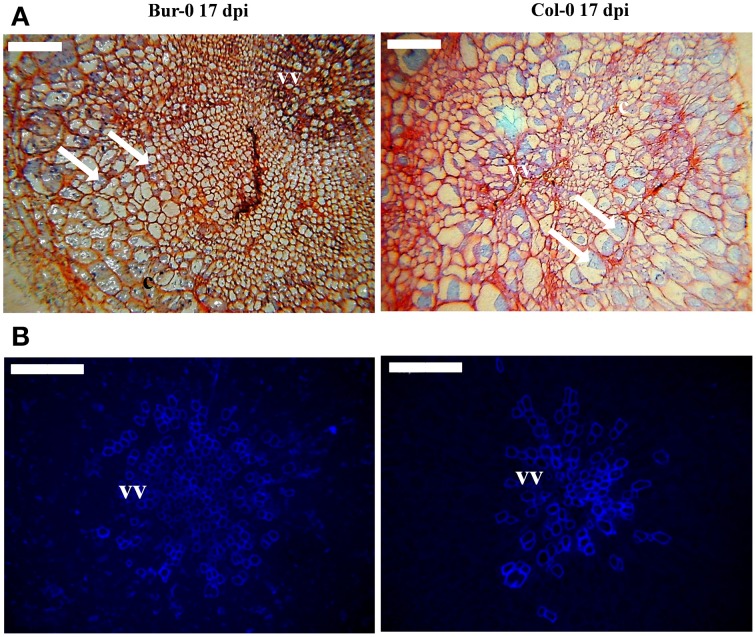
**(A)** Safranin and blue cotton and **(B)** aniline blue stained radial sections of roots infected by *Plasmodiophora brassicae* in the partially resistant accession Bur-0 and the susceptible accession Col-0. Roots were sampled at 17 dpi, then fixed and immobilized in resin as described in Materials and Methods part. **(A**,**B)**, Histological sections were cut with a microtome and stained in cotton blue and safranin to visualize pathogen plasmodia (colored in blue in **A**), plant cell walls (colored in red in **A**), and vascular vessels colored by aniline blue in **(B)** respectively. White arrows indicate plasmodia structures in infected cells. For each condition, the image shown is representative of the observations performed on at least six independent root samples. Annotations: vv, vascular vessels; c, cortex. The scale bars indicate 100 μm.

Intracellular secondary plasmodia of *P. brassicae* were visualized using cotton blue and safranin staining of sections of infected Col-0 and Bur-0 roots. At 14 dpi, the outer cortex layer in both genotypes showed enlarged and disorganized cells, which are characteristic of clubroot infection. At this time point, however, the disorganization and hypertrophy of stele cells appeared to be more pronounced in Col-0 than in Bur-0, and plasmodia in central cylinder cells were smaller in Bur-0 than in Col-0 (data not shown). At 17 dpi, infected Col-0 roots displayed maximal stele cell hypertrophy associated with a highly reduced and disorganized vascular system. In comparison, infected Bur-0 roots showed lower levels of cellular hypertrophy and weak disorganization of vascular tissues (Figures 6A,B).

### Bur/Col allelic variation at the resistance QTL *PbAt5.2* was associated with the levels of both clubroot camalexin response and *P. Brassicae* growth

We previously showed that QTL *PbAt1* and *PbAt5.2* are the two genetic regions which mainly contribute to the quantitative partial resistance in Bur-0 (Jubault et al., 2008). The objective was then to establish whether the presence of the Bur-0 allele in the *PbAt1* and *PbAt5.2* regions is associated with the high levels of camalexin accumulation in Bur-0. We used two pairs of Heterogeneous Inbred Families (HIF) lines derived from the Bur-0 × Col-0 RIL lines 499 and 508 (cf Materials and Methods). The HIF paired lines 499-Col and 499-Bur have an identical homozygous genetic background issued from the recombination between the Col-0 and Bur-0 genomes (Supplementary Figure S2), except in the genomic region between markers c5_14766 and c5_21319. Between those two markers, which comprise the confidence interval of QTL *PbAt5.2*, 499-Col, and 499-Bur carry either the Col-0 or Bur-0 alleles in the homozygous state, respectively (Supplementary Figure S2). Comparison of the phenotypic behaviors of 499-Col and 499-Bur near isogenic lines thus allowed the effect of the Bur-0/Col-0 allelic variation at QTL *PbAt5.2* to be tested. Similarly, the two other lines, 508-Col and 508-Bur, have the same homozygous recombinant genetic background (different from the genetic background of the 499 line) derived from both the Col-0 and Bur-0 genomes, and differ only from each other in the QTL *PbAt1* region, i.e., between markers c1_00593 and c1_08385 (Supplementary Figure S3). Comparison of the phenotypic behaviors of 508-Col and 508-Bur allowed the phenotypic consequences of the Bur-0/Col-0 allelic variation at the QTL *PbAt1* to be evaluated (Supplementary Figure S3).

**Figure 7:**
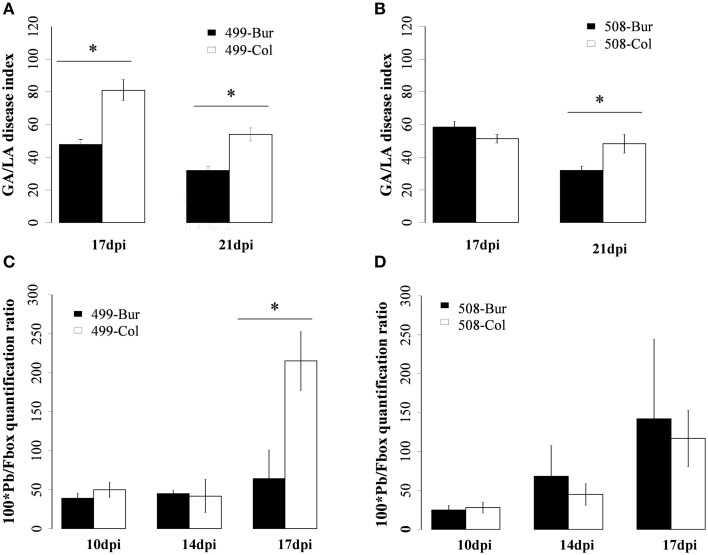
**Clubroot symptoms and accumulation of DNA from *Plasmodiophora brassicae* in infected roots of the HIFs 499 and 508**. 499-Bur and 499-Col harbor the Bur and Col alleles, respectively, at the QTL *PbAt5.2*. 508-Bur and 508-Col harbor the Bur and Col alleles, respectively, at the QTL *PbAt1*. **(A,B)** Clubroot symptoms evaluated using the GA/LA disease index from image analysis as described in the Materials and Methods Section. Error bars represent standard error (Four independent biological replicates, 18 plants per biological replicate). **(C**,**D)** Pathogen DNA quantification (Pb) by qPCR after normalization with the *Fbox* gene from *Arabidopsis*, at 10, 14, and 17 dpi (Three independent replicates, 12–54 plants per biological replicate). Asterisks indicate statistically significant differences according to the Wald tests applied on a linear mixed model (*P* < 0.05).

We first validated the effect of QTL *PbAt5.*2 and *PbAt1* on clubroot and pathogen development at 17 and 21 dpi in these two HIF lines (Figure 7). In the HIF lines 499, the Col-to-Bur allelic substitution (in the genomic regions of *PbAt5.2*) significantly reduced the severity of gall symptoms (Figure 7A) and conferred a one third reduction of pathogen growth at 17 dpi (Figure 7C). In contrast, the allelic substitution at *PbAt1*, between 508 HIF lines, significantly reduced clubroot symptoms (Figure 7B) but did not have an impact on pathogen growth (Figure 7D), suggesting that different genetic factors may control gall development and pathogen growth.

**Figure 8:**
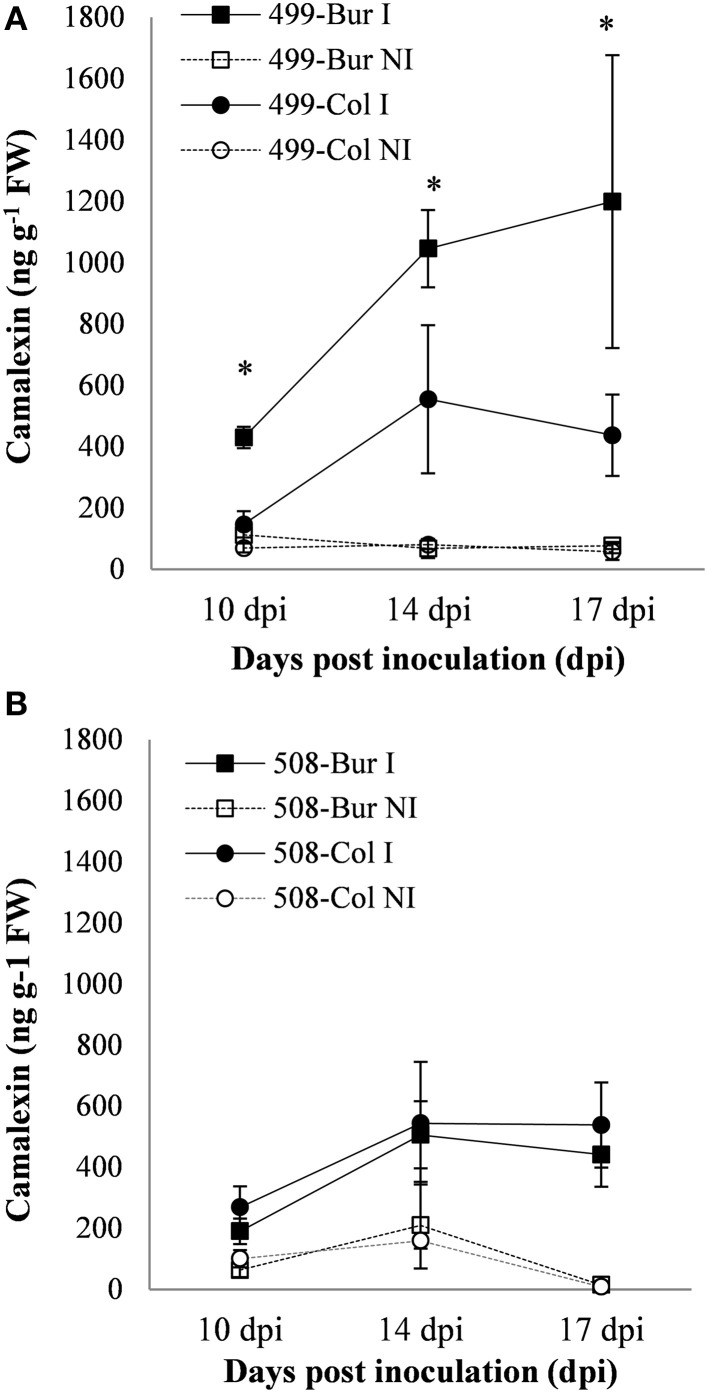
**(A,B)** Camalexin content in infected (continuous lines) and non-infected (dashed lines) roots of the HIFs 499 **(A)** and 508 **(B)** at 10, 14, and 17 dpi. 499-Bur and 499-Col harbor the Bur-0 and Col-0 alleles, respectively, at the QTL *PbAt5.2*. 508-Bur and 508-Col harbor the Bur-0 and Col-0 alleles, respectively, at the QTL *PbAt1*. **(A**,**B)**, Camalexin was quantified in root methanol extracts using UPLC-MS/MS, and is expressed as ng g^−1^ of the fresh weight. Error bars represents standard error (Three biological replicates, 12–54 plants per biological replicate). Asterisks represent statistically significant differences according to the Wald tests applied on a linear mixed model (*P* < 0.05).

The camalexin content was then analyzed in inoculated and non-inoculated roots of the HIF 499 and HIF 508 pairs at 10, 14, and 17 dpi (Figure 8). During clubroot infection, camalexin levels increased significantly in both HIF pairs. Comparison of camalexin accumulation in infected HIF 499-Bur and 499-Col lines revealed that the Bur-allele at the *PbAt5.2* region leads to a significant enhancement in the amount of camalexin (Figure 8A). In contrast, analysis of the 508 HIF lines revealed that allelic variation in the *PbAt1* region did not affect the camalexin levels in response to clubroot (Figure 8B).

**Figure 9:**
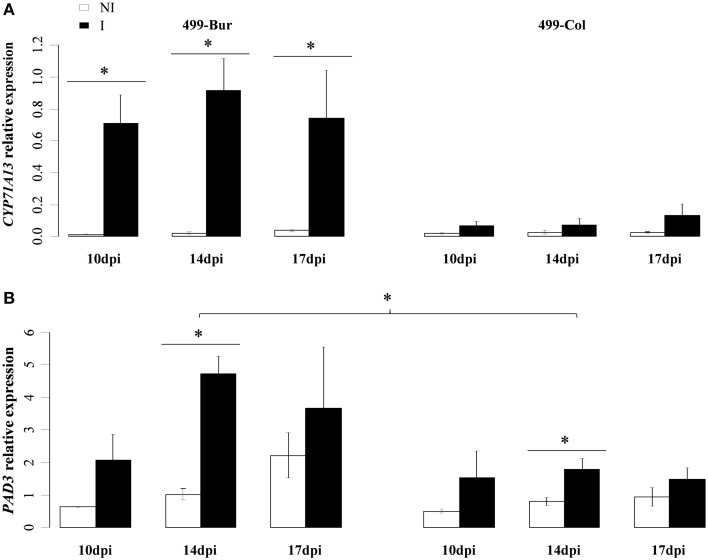
**(A)** Transcript levels of *CYP71A13* and **(B)**, *PAD3* in infected (black bars) and non-infected roots (white bars) of the HIF 499 at 10, 14, and 17 dpi. 499-Bur and 499-Col harbors the Bur-0 and Col-0 allele, respectively, at QTL *PbAt5.2*. **(A,B)**, Expression levels were normalized using the reference gene *PP2A*. Error bars represent standard error (Four independent replicates, 12–54 plants per biological replicate). Asterisks indicate statistically significant differences according to the Wald tests applied on a linear mixed model (*P* < 0.05).

We then determined the expression of camalexin biosynthesis genes *CYP71A13* and *PAD3* in the 499 HIF lines, in order to test whether the allelic variation at the QTL *PbAt5.2* affected their expression levels (Figure 9A). *CYP71A13* and *PAD3* expression was similar in non-inoculated roots for both lines. Clubroot infection did not induce *CYP71A13* expression in 499-Col, but this gene was induced in 499-Bur at all three time points (10, 14, and 17 dpi). This suggests that the Bur/Col allelic substitution in the region of the QTL *PbAt5.2* had a significant effect on the clubroot-triggered transcriptional induction of *CYP71A13* observed in the parental line Bur-0 (Figure 3B). *PAD3* was induced at 14 dpi in both 499-Col and 499-Bur, but its expression levels were significantly higher in 499-Bur, which harbors the Bur-0 allele at *PbAt5.2*, than in 499-Col (Figure 9B). Thus, in the genetic background of the 499 HIF lines, allelic variation at QTL *PbAt5.2* was linked to both clubroot-induced biosynthesis of camalexin and transcriptional regulation of *CYP71A13* and *PAD3*.

## Discussion

A preliminary screen for contrasted biochemical defense responses to clubroot between the Bur-0 and Col-0 accessions highlighted the defense-related compound camalexin as a promising marker associated with partial resistance. The initial aim of this study was to clarify the extent to which the induction of camalexin contributes to the different degrees of basal/partial resistance to clubroot in Col-0 and Bur-0.

Infection with isolate eH led to a significant level of camalexin accumulation during the secondary phase of clubroot infection in both Col-0 and Bur-0. This finding is consistent with that of Siemens et al. (2008), who reported camalexin accumulation in the Col-0 response to *P. brassicae* (isolate e3) infection, at the latest time point of infection (28 dpi). Camalexin levels were four times higher in the partially resistant accession Bur-0 than in Col-0 at the end of the kinetic time course. Thus, as camalexin levels accumulated, pathogen and/or symptom development was inhibited. Indeed, there was a clear enhancement in clubroot symptoms and pathogen development in the camalexin deficient mutant *pad3* (Col-0 genetic background). Under our laboratory conditions, this mutant exhibited more severe clubroot symptoms than Col-0 and increased pathogen development when challenged with the eH isolate. Siemens et al. (2008) previously reported that when challenged with the e3 isolate the *pad3* mutant was as susceptible as Col-0. Isolates eH and e3 are both derived from the field isolate “e,” but they showed a different pattern of pathogenicity (Fähling et al., 2003). In addition to this difference between the isolates used, the experimental conditions, the sampling time and the methods for quantifying clubroot resistance are quite different from those used in Siemens et al. (2008), and could explain the differences in the results.

Both Col-0 and Bur-0 clearly established a compatible interaction with the eH isolate, as illustrated by gall development, the detection of high concentrations of pathogen DNA during the secondary phase of infection, and the observation of secondary plasmodia. However, Bur-0 exhibited a partial resistance phenotype (fewer symptoms, less pathogen DNA and less secondary plasmodia) compared to Col-0, corroborating previous findings (Alix et al., 2007; Jubault et al., 2008). The molecular and histological data highlighted that the partial inhibition of pathogen development started in Bur-0 at 14 dpi, i.e., during the secondary phase of infection. This timing is consistent with the idea, discussed in Hatakeyama et al. (2013), that genetic resistance factors often inhibit plasmodia development during the secondary rather than the primary infection. In Bur-0, the setup of partial resistance correlated with the camalexin response. Thus, enhanced induction of camalexin biosynthesis in Bur-0, compared to Col-0, appears to contribute to its partial post-invasive resistance.

Several reports associated induction of camalexin biosynthesis with the transcriptional induction of a set of genes encoding key enzymes in the pathway. For example, Millet et al. (2010) showed that *PAD3, CYP71A13*, and *CYP71A12* expression increased in response to Flg22 treatment in *Arabidopsis*. In the present study, *CYP79B2, CYP71A13*, and *CYP71A12* were significantly more induced during the secondary phase of infection in Bur-0 compared to Col-0. Thus, enhanced camalexin biosynthesis in Bur-0 appears to be controlled at the transcriptional level through the induction of several camalexin biosynthetic enzymes. The induction of *CYP71A13* in infected Bur-0 roots was interesting, as it occurred as early as 10 dpi and was sustained all along the secondary phase of infection. This suggests that the IAN biosynthetic step plays a prominent role in the regulation of camalexin biosynthesis during clubroot infection.

Surprisingly, *PAD3* is expressed at much lower levels in Bur-0 than in Col-0 in both non-inoculated and inoculated roots. No statistically significant induction of this gene was detected in infected Bur-0 roots. This induction may have been missed for technical reasons due to the low abundance of *PAD3* transcripts in Bur-0. Nevertheless, our data suggest that a high level of *PAD3* transcription is not absolutely necessary for high levels of camalexin accumulation, at least in Bur-0. Interestingly, the HIF lines 499-Bur and 499-Col both harbor the Col-0 allele at the *PAD3* locus on chromosome 3 (Supplementary Figure S2). In addition, the *PAD3* transcript was more abundant in the non-inoculated roots of these two lines than the parental line Bur-0. This could suggest that the low level of *PAD3* transcription in Bur-0 is related to allelic variations in the coding sequence or in the regulatory sequences. As large amounts of camalexin are still biosynthesized in Bur-0 infected roots, it is possible that in this genotype a specific locus encodes an additional enzyme, which is redundant with PAD3. In this context, the transgressive behavior of 499-Bur, which accumulates even more camalexin than Bur-0, could be explained by a synergistic effect between a hypothetical *PAD3*-like locus and the Col-0 allele at the *PAD3* locus. However, we could not identify a *PAD3* homologous sequence in the Bur-0 genome [data from Gan et al. (2011), available on the 1001 genomes website: http://1001genomes.org]. Alternatively, we can speculate that the translation of the *PAD3* transcript or PAD3 protein stability is higher in Bur-0, thus explaining why low levels of *PAD3* transcription do not impair the biosynthetic flux toward camalexin. The introgression of a *pad3* mutation into the Bur-0 genetic background could be of great interest to solve this tricky question.

Our data clearly demonstrated that the Bur/Col allelic substitution in the *PbAt5.2* region (chromosome 5) drove the level of clubroot-induced camalexin biosynthesis (including the induction of key genes involved in the camalexin biosynthetic pathway) and contributed to partial inhibition of *P. brassicae* development. Together with other features discussed above (enhanced symptoms observed in the *pad3* mutant and paralleled kinetics of partial resistance and camalexin biosynthesis in Bur-0), these results give additional support to a model where camalexin levels are related to the quantitative control of clubroot infection.

In *A. thaliana*, the role of camalexin as a second layer of defense was reported to contribute to different resistance “types” such as in the case of the incompatible interaction with *Phytophthora brassicae* (Schlaeppi et al., 2010), and in the non-host interaction with *Erysiphe pisi* (Sanchez-Vallet et al., 2010). Interestingly, using a quantitative genetics approach, camalexin accumulation was also associated with several resistance QTLs toward different isolates of the necrotrophic fungus *Botrytis cinerea* (Denby et al., 2004; Rowe and Kliebenstein, 2008). Our findings provide an additional example of the role of camalexin in post-invasive pathogen inhibition in the context of partial quantitative resistance in the root, to a compatible isolate of an obligatory biotrophic pathogen.

The confidence interval of *PbAt5.2* is bordered by the genes At5G46260 and At5G47690. The interval includes several plant defense-related genes but no single gene (such as *PAD3, CYP71A12*, or *CYP71A13*) that would be directly linked with the camalexin biosynthesis pathway. Gene regulation in the HIF pair 499-Bur/499-Col suggests instead that the allelic variation in the *PbAt5.2* region is associated with a signaling process potentially controlling the expression of genes involved in all steps of the camalexin biosynthetic pathway. Substantial additional studies are now needed to identify the nature of the causal nucleotide variation(s) underlying QTL *PbAt5.2*, and to clarify which molecular mechanisms are driving the modulation of clubroot-triggered camalexin biosynthesis.

## Author contributions

SL, AG, MM contributed to initial hypotheses and to the design of the whole project. SL, CL, AG, JL, AR, and MJ contributed to the design and the conduct of clubroot assays and samplings. NM developed the analytical method for the quantification of camalexin. SL, CL, and NM contributed to all metabolite extractions and biochemical analyses. SL and JL performed PCR and RT-qPCR analyses. SL and AL did the histology work. All co-authors contributed to the interpretation of data. SL, AR, AG, and MM wrote the manuscript.

### Conflict of interest statement

The authors declare that the research was conducted in the absence of any commercial or financial relationships that could be construed as a potential conflict of interest.
