# Supplementary material for: Camalexin contributes to the partial resistance of Arabidopsis thaliana to the biotrophic soilborne protist Plasmodiophora brassicae
Source: Front Plant Sci. 2015 Jul 21;6:539. doi: 10.3389/fpls.2015.00539 (PMC4508518; doi:10.3389/fpls.2015.00539)
Supplement: Supplementary file 1 [file DataSheet1.PDF]

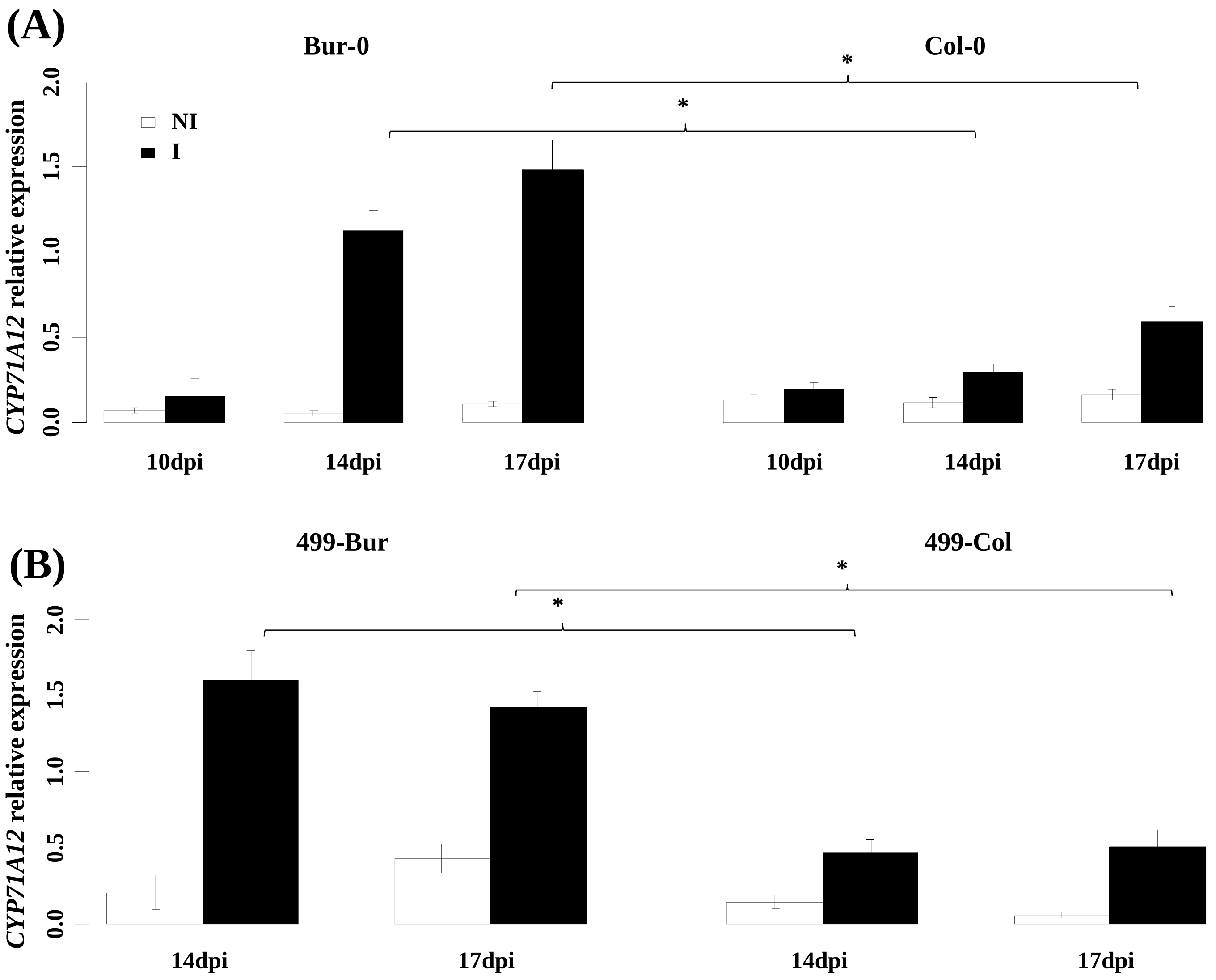

Supplementary Figure S1 Transcript levels of *CYP71A12* in infected (black bars) and non-infected roots (white bars) of the parental lines Col-0 and Bur-0 at 10, 14 and 17 dpi and in the HIF 499 at 14 and 17dpi. 499-Bur and 499-Col harbours the Bur-0 and Col-0 allele, respectively, at QTL *PbAt5.2*. **(A)** and (**B**), Expression levels were normalized using the reference gene *PP2A*. Error bars represent standard error (6 biological replicates, 12 to 24 plants per biological replicate). Asterisks indicate statistically significant differences according to the Wald tests applied on a linear mixed model ($P<0.05$).
