# Supplementary figures and images for: Camalexin contributes to the partial resistance of Arabidopsis thaliana to the biotrophic soilborne protist Plasmodiophora brassicae
Source: Front Plant Sci. 2015 Jul 21;6:539. doi: 10.3389/fpls.2015.00539 (PMC4508518; doi:10.3389/fpls.2015.00539)

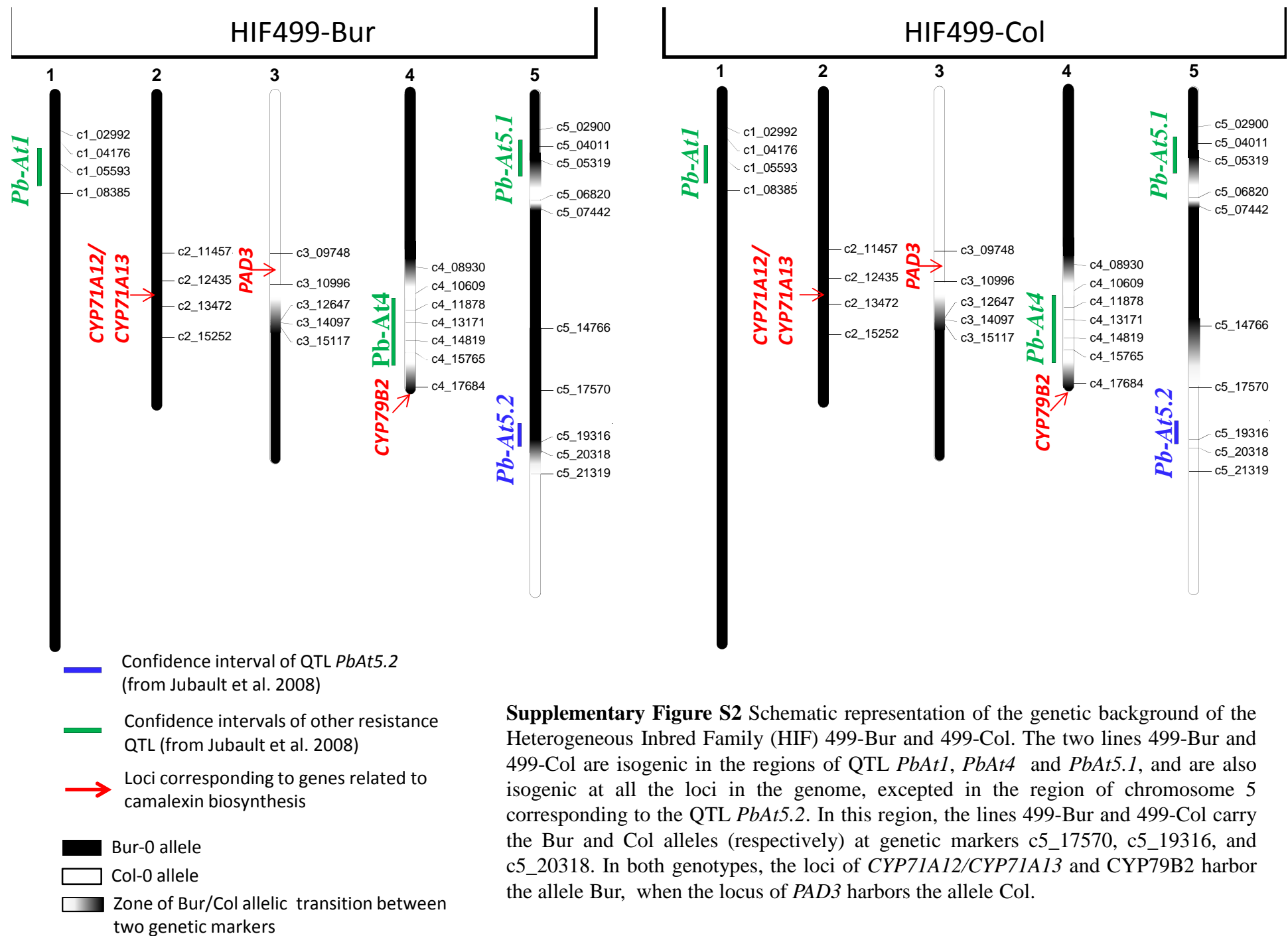

Supplement: Supplementary file 2 [file DataSheet2.PDF]
