# Supplementary material for: Camalexin contributes to the partial resistance of Arabidopsis thaliana to the biotrophic soilborne protist Plasmodiophora brassicae
Source: Front Plant Sci. 2015 Jul 21;6:539. doi: 10.3389/fpls.2015.00539 (PMC4508518; doi:10.3389/fpls.2015.00539)
Supplement: Supplementary file 3 [file DataSheet3.PDF]

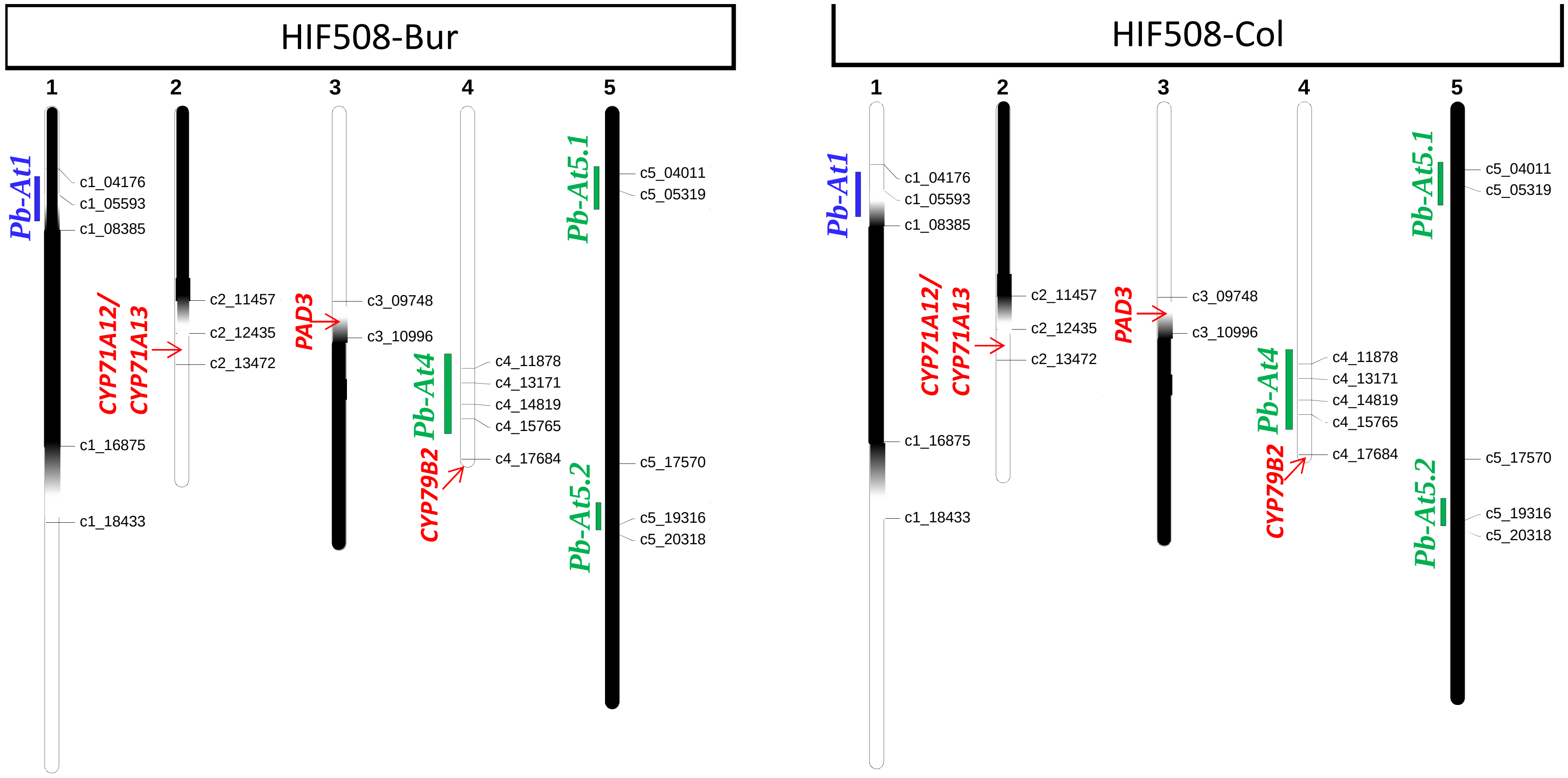

Confidence interval of QTL *PbAt1* (from Jubault et al. 2008)

Confidence intervals of other resistance QTL (from Jubault et al. 2008)

Loci corresponding to camalexin biosynthesis genes

Bur-0 allele

Col-0 allele

Zone of Bur/Col allelic transition between two genetic markers

Supplementary Figure S3 Schematic representation of the genetic background of the Heterogeneous Inbred Family (HIF) 508. The two HIFs 508-Bur and 508-Col are isogenic at QTL *PbAt4*, *PbAt5.1* and *PbAt5.2*, and at all the loci in the genome, excepted in the region QTL *PbAt1* (chromosome 1). In this region, HIF 508-Bur and HIF 508-Col carries the alleles Bur and Col (respectively) at the genetic markers c1_02992, c1_04176 and c1_05593. In both genotypes, the loci of *CYP71A12/CYP71A13* and *CYP79B2* harbor the allele Col-0 and the allelic status of *PAD3* is not known.
